# Automatically Attributing Mobile Threat Actors by Vectorized ATT&CK Matrix and Paired Indicator

**DOI:** 10.3390/s21196522

**Published:** 2021-09-29

**Authors:** Kyoungmin Kim, Youngsup Shin, Justin Lee, Kyungho Lee

**Affiliations:** 1 Institute of Cyber Security & Privacy (ICSP), Korea University, Seoul 02841, Korea; richard2104@korea.ac.kr (K.K.); shinyounsup@korea.ac.kr (Y.S.); jeminjustinlee@korea.ac.kr (J.L.); 2 Center for Information Security Technology (CIST), Korea University, Seoul 02841, Korea

**Keywords:** threat intelligence, mobile security, cyber security

## Abstract

During the past decade, mobile attacks have been established as an indispensable attack vector adopted by Advanced Persistent Threat (APT) groups. The ubiquitous nature of the smartphone has allowed users to use mobile payments and store private or sensitive data (i.e., login credentials). Consequently, various APT groups have focused on exploiting these vulnerabilities. Past studies have proposed automated classification and detection methods, while few studies have covered the cyber attribution. Our study introduces an automated system that focuses on cyber attribution. Adopting MITRE’s ATT&CK for mobile, we performed our study using the tactic, technique, and procedures (TTPs). By comparing the indicator of compromise (IoC), we were able to help reduce the false flags during our experiment. Moreover, we examined 12 threat actors and 120 malware using the automated method for detecting cyber attribution.

## 1. Introduction

With the development of information technology, real-world values such as information assets and financial assets have begun to move into cyberspace. In particular, in the post-PC era [1] after 2000, mobile devices and IoT devices are frequently used in people’s daily lives, and various values are stored more in mobile devices than in PCs. The explosive proliferation of mobile devices exposes individuals and organizations to the risks of being hit with multi-vector cyber attacks. With the emergence of organized threat actors targeting these assets and carrying out sophisticated cyberattacks, they began to launch a variety of threats targeting mobile devices. Threats arising from mobile devices are the same as threats from PCs. Installing malicious content can infect your smartphone with worms, Trojans, or other types of viruses that compromise your security and privacy or take complete control of your device. The difference is that such malicious content can spread more easily due to the development of mobile network technology that provides a continuous Internet connection function through a 3G or Wi-Fi network to a smartphone [2]. Because of these characteristics, mobile attacks are considered an attractive attack vector from the attacker’s point of view. Mobile attacks were carried out in various forms, from mobile sensors to application-level malware, including attack vectors occurring in traditional PCs and servers. There have been studies on mobile malware detection and classification [3,4] and mobile threat assessment [5,6,7] to solve various mobile security threats, but there are few studies on cyber attribution that track mobile attacks and identify the attacker.

For mobile threats, it is difficult to identify the attacker for the following reasons: (i) high accessibility, (ii) various initial access, and (iii) jurisdictional limitation mobile attacks are relatively low-entry and high-reward, making them more accessible, so there is a high possibility that more diverse attackers, from nation-state actors acting for national interest to individuals, will exist. In a mobile environment, attacks are performed through diverse routines. In addition to traditional PC infection methods, various infection routes, such as infecting malicious applications in the play store, exist. Since a law enforcement agency has to undertake an investigation that crosses borders, jurisdictional limitations can hinder attribution in cross-border cybercrime investigations. Official channels are needed to request help, which impedes the progress of collecting cyber evidences; however, it is crucial to attribute threat actors in the mobile environment. This is because threat actors freely move between PC and mobile environments to achieve their goals. Furthermore, if the attack procedures for each threat actors in the mobile environment is established and stored, and can be connected to the attribute threat actor in the existing PC environment, it will be possible to improve the understanding of the current cyber threat environment to a high level and to materialize the countermeasures. The traditional cyber attribution method was considered to attribute the mobile threat. In order to perform cyber attribution [8,9], a complex evaluation that considers both technical and socio-political is included. At this time, when checking the technical indicators required for attribution, the tactic, technique, and procedure (TTP) of the attack are analyzed and the indicator of compromise (IoC) is compared. These methods, however, are managed by cyber threat intelligence experts to collect, share, and manage data manually; therefore, errors may occur when analyzing numerous malware and incidents manually.

This paper presents a methodology to classify and attribute the attack background of mobile malware with known threat actors through automated TTP and IoC analysis. For automated TTP analysis, we present two methods: mathematical modeling of the ATT&CK matrix and IoC pairing to avoid false flags. Through this, it shows the benefit of quickly organizing the TTPs of numerous mobile incidents.

## 2. Related Works

### 2.1. Status Quo of Cyber Attribution

In general, cyber attribution is performed to identify the threat actors and classify the attack group, which includes: (i) programming techniques (code similarity), (ii) attack tactics and strategies, (iii) infiltration target fields, (iv) base infrastructure (IP, domain), and (v) carrying out an in-depth investigation taking into account geographic characteristics and language habits.

State agencies such as US CISA and cyber threat intelligence companies such as FireEye, CrowdStrike, Kaspersky, and Microsoft Security Intelligence define national threat actors and track and share their activities when a cyber incident occurs. According to the “*Guide to Cyber Attribution*” [10], the US Information Community defines the attackers through key indicators such as tradecraft, infrastructure, malware, attack intention, geopolitical situation, and external information.

**Figure 1:**
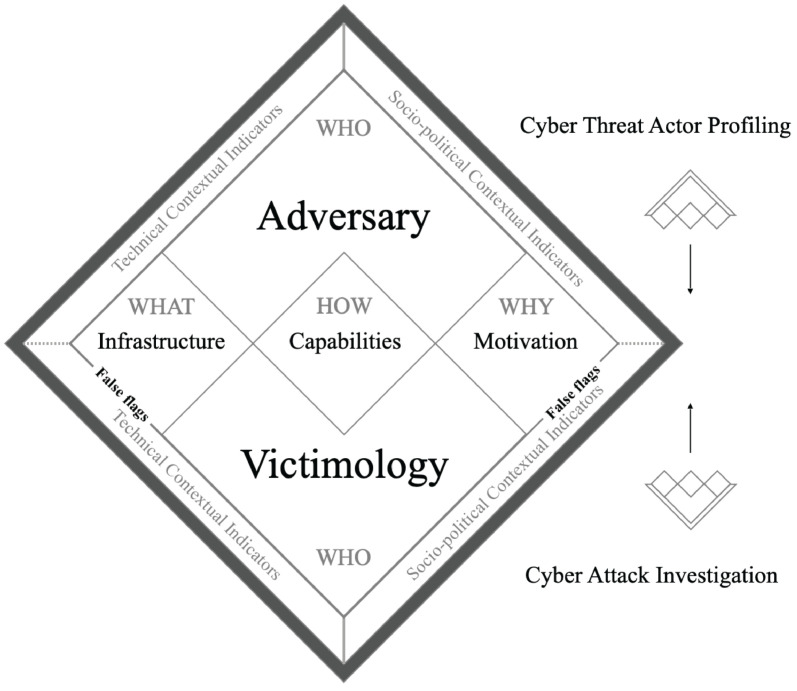
Cyber attribution model (CAM) presented by Pahi is a method that identifies the attacker [11]. CAM helps with the cyber threat actor profiling from the adversary group. It also helps with the cyber attack investigation.

Among the various studies that refined cyber attribution, Pahi [11] proposed the cyber attribution model (CAM). As shown in Figure 1, he expressed that cyber attribution is possible by conducting cyber threat actor profiling from the adversary and cyber attack investigation from the victim. This study said that complex associations and false flags could be identified if technical and socio-political contextual indicators were used in combination.

In order to consider the socio-political contextual indicator, it is necessary to focus on the motivation and circumstances of the attacker. However, this part is difficult to express quantitatively, and if the attacker is an individual, not a state-based entity, it will be difficult to understand the motivation or to classify it as a limited purpose; they also limited the scope of cyber attacks to professional and organized cyber attacks.

The studies described above did not talk about mobile-specific cyber attribution [11]. Obviously, there is a difference between the PC breach index and the breach index that can occur on mobile. Due to differences in development platforms, the APIs used are different, and different indicators of infringement are extracted as the simplified code is used. To solve these problems, we introduce the cyber attribution methodology that applies TTP and IoC specialized for mobile.

### 2.2. Technical Attribution Methods for Mobile Threats

Technical attribution studies have also been conducted in mobile circumstances. Silva Sebastian [12] presents a novel approach for identifying identical operations within mobile app markets: developer accounts and other indicators of compromise (IoCs) belonging to the same owner. According to the study,

The methodology discovers at least one previously unknown developer account in 94% of the operations. Furthermore, it reveals that operations that look dead still have active developer accounts.

However, while this methodology is effective to track the same developer in the official mobile application market, we discuss how to attribute and classify collected mobile malware regardless of where it was collected.

### 2.3. ATT&CK Matrix

The MITRE’s ATT&CK Framework is the most widely known and utilized methodology for expressing the activity of a cyber attack or threat actor [13,14]. The MITRE’s ATT&CK Framework analyzes the activities of cyber attacks and threat actors from the perspective of TTPs (tactics, techniques, and procedures), and composes and expresses them in the form of ATT&CK matrix. MITRE receives quite a wealth of information because data are referenced from several vendors, such as Kaspersky, ESET, and Trend Micro.

**Figure A1:**
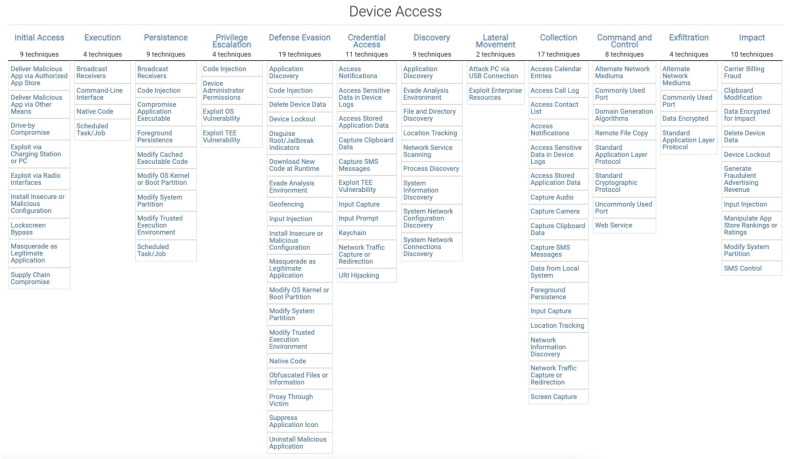
The ATT&CK matrix for the mobile OS consists of two parts. The first part of the matrix covers the device access.

**Figure A2:**
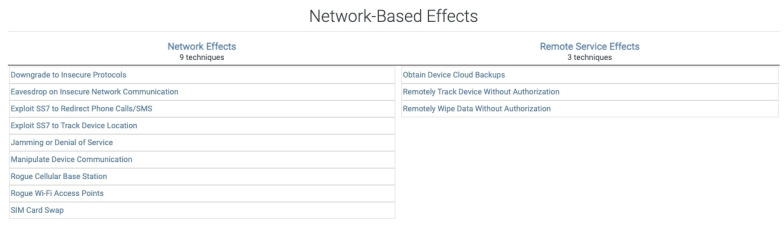
The ATT&CK matrix for the mobile OS consists of two parts. The second part of the matrix covers the network-based effects.

There are three versions of this ATT&CK matrix: Enterprise, Mobile, and ICS. The most common enterprise framework has 14 tactic procedures from reconnaissance to impact, and 185 attack techniques to be used for each tactic are organized. There are detailed sub-techniques in each technique so that 367 sub-techniques can express one attack incident in detail. Each tactic and technique used in one case is colored in a matrix, and the TTP overview can be seen with this matrix alone. On the other hand, mobile ATT&CK consists of only 14 tactics and 89 techniques, and sub-techniques are not separately organized. As shown in Figure A1 and Figure A2, the 14 tactics of the mobile ATT&CK matrix are divided and utilized in the Device Access stage and Network-based Effects stage.

Since these MITRE ATT&CK matrices provide the most consequential TTP analysis for APT group classification, we introduce the cyber attribution methodology using this matrix.

### 2.4. Automated Malware Analysis

Several automated malware analyzers have been developed to analyze malware and collect various indicators of compromise quickly [15,16,17]. They work in a virtual machine called a sandbox that performs dynamic analysis and infects them. Popular sandboxes include the Joe sandbox [15], Intezer [16], and Cuckoo sandbox [17].

Intezer performs the analysis using the `DNA mapping’ technology called Code Intelligence™. It splits a file or binary into thousands of fragments, divides it into billions of code fragments, and compares it to Intezer’s Code Genome Database. Code Intelligence™ is known as a famous sandbox for detecting code reuse and code similarity. In particular, Joe sandbox provides integrated and precise malware analysis for various operating system executable files such as Windows, Linux, Mac, Android, and iOS. In addition, the mobile environment supports the MITRE ATT&CK sub-technique, a standard framework using each intrusion threat model, to map threat behavior signatures and provide proper response procedures to security analysts. Joe sandbox offers SaaS (Software-as-a-Service) and on-premise versions. This paper uses the Joe sandbox Ultimate on-premises version to collect the ATT&CK matrix in an automated methodology.

## 3. Methodology

**Figure 2:**
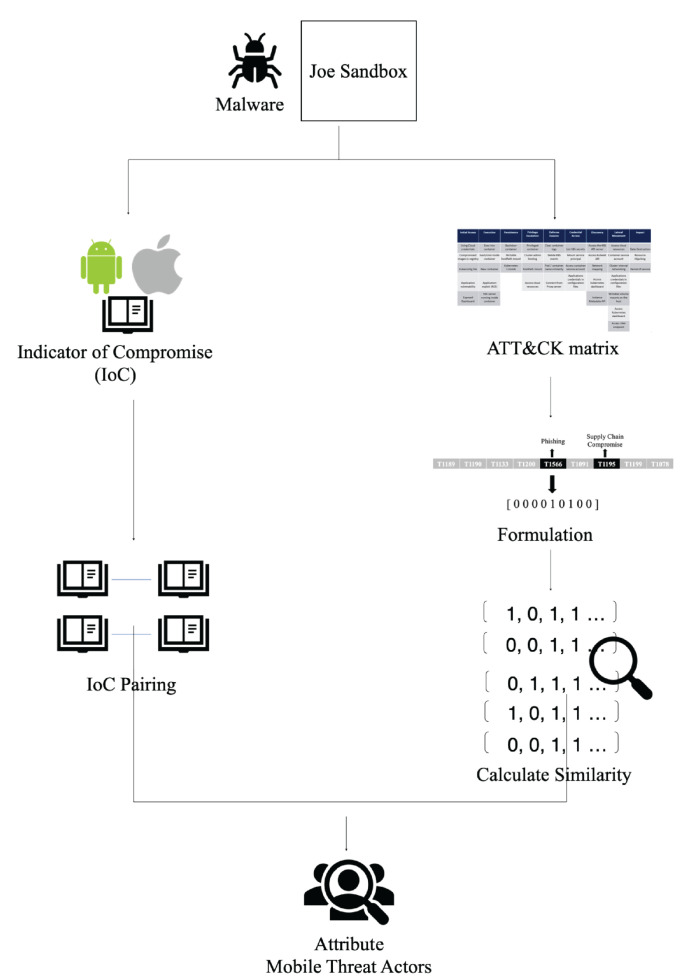
The methodology proposed in this study consists of two parts. The two parts are the mathematical modeling of ATT&CK matrix and IoC pairing.

Our study introduces a methodology that automatically enables mobile APT group attribution. The goal of this study is to provide a new indicator for cyber attribution against large-scale mobile threats through automation. The proposed methodology is divided into two main ideas shown in Figure 2 The first part of the methodology utilizes and collects the mathematically modeled ATT&CK matrix for quantified TTP collection, and the second part compares the IoC pairs considering the false flag.

First, when mobile malware is collected, the ATT&CK matrix is extracted through Joe sandbox. Joe sandbox has a function that automatically creates a possible MITRE’s ATT&CK based on various signatures from the 2018 Fire Opal version. Currently, both “.ipa” files for ios and “.apk” files for Android are available for ATT&CK analysis. The obtained ATT&CK matrix is vectorized through mathematical modeling and the vectorized matrix is stored. Each vectorized matrix representing one TTP per one malware is calculated.

TTP is also the main information used for cyber attribution, but more clear and detailed information is needed. Considering IoC for this is the second step. The collected malware collects several IoCs from not only sandbox but also in public malware intelligence system such as Virustotal [18]. In particular, the method collects IoC used for various malware classification or attribution studies as candidates.

If only a single indicator is considered, it is not easy to identify a false flag intentionally assigned by a threat actor, so it is important to find a combination that can provide a strong basis for assisting attribution. The task of finding such a combination can be called indicator pairing.

In summary, after comparing the TTPs collected through the ATT&CK matrix, IoC among the candidates is compared.

### 3.1. Mathematical Model of ATT&CK Matrix

By employing ATT&CK, the procedures, techniques, and tactics can be observed; however, mathematical modeling was required to utilize this table form in the automated attribution model.

**Figure 3:**
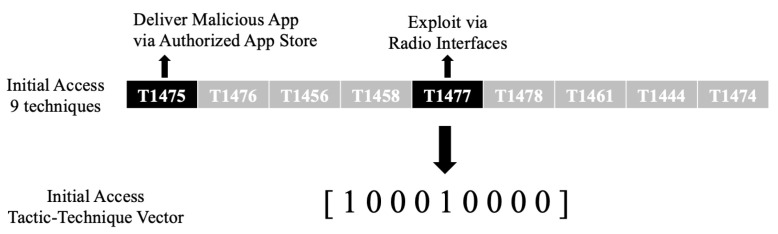
The ATT&CK matrix helps create a common language for the adversary group’s attack behavior. Our study vectorized the tactics and techniques from the APT groups.

A row of Mobile ATT&CK matrix consists of 14 tactics, and each tactic consists of multiple techniques. We convert the ATT&CK matrix into a tactic unit vector form as shown in Figure 3 to make it easy for similarity analysis. In the ATT&CK matrix, the used technique is expressed as 1, and the unused technique is expressed as 0; therefore, the ATT&CK matrix expressing one malware event is expressed as a vector set consisting of 14 tactic vectors.

This is expressed as: For malware X, a set of 14 Tactic–Technique vectors is assumed to be $T_{x}$. $T_{x}$ consists of 14 tactic $T_{i}x$ vectors, and their elements consist of 0 and 1. $T_{x}$ is an integrated vector set of $T_{i}x$ vectors.

If it can be expressed as a vector set in this way, it is possible to measure the TTP similarity of the collected malware.

(1) $$sim\left(T_{x},T_{y}\right)=\frac{T_{x}\cdot T_{y}}{∥T_{x}∥∥T_{y}∥}$$

### 3.2. Indicator of Compromise Pairing

Although it was possible to collect and store the TTP of large-scale malware through the quantified ATT&CK matrix in the previous step, it can identify the approximate TTP usage method, but it is still insufficient information to conduct attribution.

To make more precise technical attribution, information on IoCs should be utilized.

However, some attackers arbitrarily manipulate the attack indicators to disguise themselves as other threat actors or leave a false flag that transfers responsibility. Therefore, the false flag should be considered when collecting IoC. Although it is impossible to identify the smoking gun in cyber attribution, it is possible to partially solve the problem by considering the causes of false flags.

Skopik [19] explained the reason why the false flag occurs as follows:

- Exploits work in the past and use recycled code/variants of previous attacks that have been disclosed.
- Exploits are developed to mimic the behavior and complexity of known malware.
- Exploits and malware are purchased, not developed.
- An attack is rented as a service.
- Malware connects to known C&C infrastructure, but is not designed by server operators.
- The C&C server uses a third-party infrastructure that is not affiliated with the attacker.
- Breach is socially designed to mislead investigations into other operators.
- Executing additional malicious actions hides your true intention to mislead investigators.

The most significant cause of the false flag is that the malicious code is provided in recycled code and service. To solve this problem, the method of comparing with the past event based on the string in the code area should not be considered. In addition, it is necessary to closely examine the characteristics between IoCs to know what IoCs are difficult to modify, and not only view individual IoCs but to compare them with related IoCs.

In this study, (i) false flag and (ii) IoC specialized for mobile is considered using the indicator pairing method.

The indicator pairing method is as follows. After attribution forms a set of known malware for the same threat actor, a method is used to obtain a pair of indicator types that the malware have in common. For example, after finding the correlation between indicator types for malware of the same threat actor, IoC pairs with high correlation are identified; if a high correlation is formed between the same IoC pair in malware of other threat actors, it can be said that this IoC pair is properly paired.

This method can be used only when malware used by various threat actors is identified and the same IoC pair is configured. If an IoC pair is found only in malware of a specific threat actor, this can be considered weak pairing. The problem is, it cannot be used if the attribution is not performed proactively for as many kinds of malware as you need.

We conducted the stored IoC pairing by covering all the heuristic utilization methods of malware researchers through previous studies. This could produce the same result as in Table 1.

In this way, when pairs of related IoCs are designated and compared in pairs, even if there is one arbitrarily manipulated individual IoC, it is not judged as a similar index. These IoCs are initially collected through Joe sandbox or VirusTotal. The similarity is measured based on the number of identical IoC pairs by first searching for IoC pairs collected from malware.

## 4. Experiments

We utilized the method of calculating false positives and precision to ensure that the methodology was correct. Two experiments were performed: (i) An experiment of only the TTP similarity of the malware, that is, a method of attributing by comparing the ATT&CK matrix, and (ii) an experiment comparing the ATT&CK matrix with the IoC pair were confirmed.

Experiments were conducted on 120 mobile malware, including 88 mobile malware collected through public information and 32 samples collected privately. The undisclosed samples are obtained in collaboration with several cyber threat intelligence firms or through self-collected information. This 120 malware are labeled data, which means a threat actor is known for each malware. These are classified into 12 threat actors, which consist of groups such as North Korea APT group Konni [20] and China Yanbian Gang [21].

The experiment is as follows. By comparing the cosine similarity of 120 ATT&CK matrix, a node connection is made between malware that exceeds the similarity threshold. The distance was determined according to the degree of similarity, and 12 clusters were obtained through the k-means algorithm. The precision and recall values were calculated by comparing the 12 groups obtained through the experiment and the labeled data.

At this time, the desired precision and recall values for the experiment are as follows.

For target Threat Actor A to be analyzed:

- TP: The malware attributed as Threat Actor A is classified as Threat Actor A.
- FP: The malware attributed as another Threat Actor is classified as Threat Actor A.
- TN: The malware attributed as another Threat Actor is not classified as Threat Actor A.
- Precision: The percentage of malware classified as Threat Actor A by the methodology that is actually Threat Actor A.
- Recall: The ratio of malware classified as threat actor A by the methodology among actual Threat Actor A.

Since the degree to which known TTP is used may differ for each threat actor, TP and FP are likely to differ significantly in the same methodology; therefore, in order to accurately calculate the TP and FP of the methodology, the average cyber-attribution ability was verified through the mean values of the precision and recall values of all threat actors.

Table 2 showed that the TTP analysis has a great effect on the classification of the attacker with fairly high precision and recall values, but higher values are required. So, we compared the experiment with IoC pairing.

The similarity of IoC pairings was calculated based on the number of IoC pairings as described above. A new experiment was needed to perform a new grouping by applying a new feature called IoC pairing to k-means clustered groups through the existing ATT&CK matrix similarity. As a result of checking how many IoC pairs are identical to examine the usefulness of only actual IoC pairing in the labeled data, we found that on average 1.78 pairs of actual malware are identical. This average value can be an indicator that can be assumed to be the same attacker when at least 1.78 pairs of IoC are identical.

However, this number is a figure when only IoC pairing feature is applied to the methodology and cannot be a standard for direct clustering. Therefore, we selected the strictness of the number of pairings to use the new criterion to clustering applied by TTP analysis. An experiment was conducted to put the number of pairs into the same group only when the number of IoC pairings among malware attribute to the ATT&CK matrix was equal to or more than *N*. *N* is the number of pairs that must be equal to be classified into the same group. In this case, the precision and recall value could be seen as shown in Table 3.

Through this, it can be seen that meaningful results are obtained for malware that exists at least one of the groups divided by the ATT&CK matrix; however, when the group is divided according to strict criteria, such as when the number of pairs N is 3 or more, the accuracy was significantly reduced.

## 5. Discussion

The experimental results described above yielded the following expected effects. First, it showed the possibility of automatic cyber attribution in a mobile environment. Based on the existing manual cyber attribution method, vectorization is included to automate, and when grouping is performed by limiting the number of appropriate IoC pairings for APT groups, a precision of 0.9148 could be obtained. Second, we quantified the mobile TTP analysis, which was expressed only qualitatively, and quantified and explained that the TTP analysis actually affects mobile cyber attribution. After vectorization of the ATT&CK matrix, the similarity of TTP could be obtained, and it was confirmed that the precision value of 0.8178 was quite helpful in identifying threat actors only with TTP analysis.

However, there is a limit on the number of samples, and the sample was tested with 12 threat actors, but as the threat actor cluster increases, there is a possibility that the accuracy will decrease. Therefore, it is necessary to find a way to improve the accuracy among many APT groups by developing the type of IoC pair through research.

Moreover, the methodology has real-world potential and can be used in cyber command and several threat intelligence agencies for large-scale cyber attribution operation. It is expected that malware researchers can greatly reduce the time for attributing mobile threat actors, and if accuracy is improved, analysis of large-scale malware incidents is possible.

## 6. Conclusions

This study explained two new ideas, the utilization of a vectorized mobile ATT&CK matrix and the indicator pairing technique while presenting a methodology for automatic cyber attribution.

By automatically formulating the mobile ATT&CK matrix in a cyber attribution method through research, it will enable automated TTP classification for many mobile threats. In addition, by providing TTP and IoC pairs to the analyst, it is possible to classify and provide mobile attacker candidates.

For future works, non-heuristic research on IoC pairing through a more quantitative methodology for IoC pairing is needed, and it needs to be applied to more samples for verification. The reason these two problems could not be solved in this study is because of the small number of samples. This is because it takes too much time to obtain labeled malware by completing one incident and cyber attribution of malware; therefore, it is necessary to increase the number of labeled samples through the steady collection and analysis of new samples by analyzers to further the study.

## Figures and Tables

**Table 1:** This paper developed four types of indicator of Compromise(IoC) pairs to avoid false flags considering the correlation between IoCs.

Pair Number	${\mathrm{IoC}}_{1}$	${\mathrm{IoC}}_{2}$
1	C&C Platform	Domain Registration Geolocation
2	C&C IP	Past Domain that IP used previously
3	Disguised Types	Malware Types
4	APK Signing Country code	Victim Geolocation

**Table 2:** The table represents the average recall and precision values of 12 threat actors by using ATT&CK similarity clusters. All values are rounded to the second decimal places.

	ATT&CK Similarity
TP	73.2
FP	16.3
TN	22.3
FN	8.4
Precision	0.8178
Recall	0.8970

**Table 3:** The table describes the calculated precision and recall values by varying the number of IoC pairs that must be satisfied to be classified into a group. All values are rounded to the second decimal places.

	N = 0	N = 1	N = 2	N = 3	N = 4
TP	73.2	78.7	88.1	60.3	23.2
FP	16.3	18.2	8.2	12.2	36.3
TN	22.3	16.8	19.2	35.3	52.3
FN	8.4	6.5	4.5	10.4	18.4
Precision	0.8178	0.7981	0.9148	0.8317	0.3899
Recall	0.8970	0.9237	0.9514	0.8528	0.5576

## Data Availability

Data available on request due to restrictions. The data presented in this study are available on request from the corresponding author. The data are not publicly available due to cooperation with third-party.
